# The role of intelligences in teams: a systematic literature review

**DOI:** 10.1007/s11846-023-00672-7

**Published:** 2023-05-19

**Authors:** Mahboobeh Davaei, Marjaana Gunkel

**Affiliations:** 1 Management School, University of Sheffield, Conduit Road, Sheffield, S10 1FL UK; 2 Faculty of Economics and Management, Free University of Bozen/Bolzano, Universitätsplatz 1, 39100 Bolzano, Italy

**Keywords:** Emotional intelligence, Cultural intelligence, Cognitive ability, Team, Literature review, J24

## Abstract

With organizations moving towards team-based structures, there is a great interest in studying organizational teams. Using a comprehensive, thorough, and systematic literature review, this study reviews the existing studies that have contributed to the importance of intelligences in various types of teams. This study intends to structure existing research, identify its current trends, and provide an overview of recent research strands and topics on the role of intelligences in organizational teams. Searches were conducted of Web of Science and EBSCO databases, and 44 eligible studies, published in Chartered Association of Business Schools (ABS) ≥ 2-star journals, were identified. The results indicate that cognitive ability, emotional intelligence, and cultural intelligence can be considered important factors contributing to various team-related outcomes. Furthermore, the findings demonstrate a growing interest in research on global virtual teams, which is a trend that is predicted to continue. Suggestions for future research directions are discussed.

## Introduction

In the late 20th and beginning of the 21st centuries, organizations worldwide started to transform their work from individual to teamwork activities (Lawler et al. 1995), and teams became central building blocks of organizations. Teams are a collection of interdependent individuals who exist to perform organizationally relevant tasks, share one or more common goals, and interact socially (face-to-face or virtually). They are perceived by themselves and others as social entities and are embedded in one or larger social systems, like organizations (Arrow et al. 2000; Kozlowski et al. 1999; Kozlowski and Bell 2003; Salas et al. 1992; Sundstrom 1999). In the last decades, the use of teams has expanded dramatically in response to increased global competition, consolidation, and innovation, and the need for more rapid, flexible, and adaptive responses (Kozlowski and Bell 2013). Teams have proved to play a fundamental role in organizational success in a global, competitive, and changeable economy (Mathieu et al. 2006).

Traditional teams involve a relatively unchanging set of members working interdependently toward a common purpose. While globalization and advanced communication technologies are changing the teams' context and driving the need to form diverse teams with complex challenges for improving effectiveness (Webber et al. 2019). An increasing number of multinational organizations structure their work through the use of global teams (Groves and Feyerherm 2011), characterized by heterogeneity in terms of national, cultural, and linguistic elements (Zander et al. 2012), to develop competitive strategies (Gluesing and Gibson 2004). Global teams can be collocated, virtual, or a combination of both. While collocated or face-to-face teams are located in close physical proximity, virtual teams, also known as ‘geographically dispersed teams’, ‘distributed teams’, or ‘remote teams’, ‘use technology to varying degrees in working across locational, temporal, and relational boundaries to accomplish an interdependent task’ (Martins et al. 2004: 808). Global teams, which operate virtually, form global virtual teams (GVTs). Also called ‘multinational and multicultural distributed teams’ (Connaughton and Shuffler 2007), and ‘transnational teams’ (Earley and Mosakowski 2000), GVTs have been conceptualized as ‘temporary, culturally diverse, geographically dispersed, and electronically communicating work group(s)’ (Jarvenpaa and Leidner 1999: 792). GVTs enable organizations to respond quickly to changing business environments (Mulebeke and Zheng 2006) and react faster to increased competition (Hunsaker and Hunsaker 2008; Pauleen 2003), thus, leading to higher effectiveness and efficiency (May and Carter 2001) and better organizational outcomes (Gaudes et al. 2007; Piccoli et al. 2004).

Despite the numerous benefits that teamwork provides for organizations, using teams is most often paired up with challenges. Given the challenges caused by different forms of teams, such as distributed membership, shorter spans of working together, or greater cross-cultural contact, the team's outcomes are influenced by various input factors. One of the important predictors of team effectiveness is the team members' collective general cognitive ability (IQ), which includes aptitudes such as reasoning, problem-solving, dealing with abstract concepts, and complex problem-solving (Gottfredson 1997). Previous research shows that team members' average IQ is related to team performance (e.g., Barrick et al. 1998; LePine et al. 1997; Neuman and Wright 1999; Tziner and Eden 1985). However, just having a number of individuals with high IQ may be useful, but it is certainly not sufficient for creating a well-functioning work group. According to Gardner's *theory of multiple intelligences* (1983), beyond general cognitive ability, there are other intelligences crucial for successful teamwork (Yost and Tucker 2000). Gardner (1983) proposed that human intelligence is pluralistic rather than unitary, and individuals possess eight relatively autonomous intelligences, including verbal-linguistic, logical-mathematical, visual-spatial, musical-rhythmic, bodily-kinesthetic, naturalistic, interpersonal, and intrapersonal.

The social aspects of intelligence may be as important, if not more important, than the cognitive aspects (Sternberg and Grigorenko 2006). Thorndike (1936) introduced the idea of social intelligence as a single concept, but later Gardner (1983) defined social intelligence as personal intelligences based on two aspects: intrapersonal intelligence and interpersonal intelligence. Personal intelligences have been suggested as an important attribute required for successful teamwork (Tarricone and Luca 2002). Interpersonal intelligence refers to the ability to understand the intentions, motivations, and desires of other people and, consequently, work effectively with them. While intrapersonal intelligence is the ability to understand one's own emotions, ideas, motivations, and self-reflection and to use such information effectively in regulating one's own life (Gardner 2006).

After the evolution of social intelligence, other related constructs have appeared. The past decades have seen increasing interest in the role of emotion in the workplace, punctuated by several important theoretical advances, such as Goleman's (1995, 1998) and Salovey and Mayer's (1990) conceptualization of emotional intelligence (EQ). EQ refers to ‘the ability to monitor one's own and other's feelings and emotions, to discriminate among them, and to use this information to guide one's thinking and actions’ (Salovey and Mayer 1990: 189). The concept of EQ is grounded in social intelligence (Cartwright and Pappas 2008; Salovey and Mayer 1990; Wong and Law 2002) and is akin to what Gardner (1983) has termed as personal intelligences (Cartwright and Pappas 2008; Gardner 2002; Salovey and Mayer 1990). EQ is an emotion-oriented part of personal abilities and is increasingly being promoted as being necessary for successful teamwork (Yost and Tucker 2000). EQ has been proven to influence team processes, like intragroup conflicts (Ayoko et al. 2008; Davaei et al. 2022; Lee and Wong 2017), team climate (Davaei et al. 2023), intra-team trust (Barczak et al. 2010; Chang et al. 2012), and team member collaboration (Cole et al. 2016), as well as team effectiveness, such as team performance (Jamshed and Majeed 2019; Lee and Wong 2017; Paik et al. 2019; Xiang et al. 2016), team cohesion, and innovation (Lee and Wong 2017).

However, EQ is culture-bound (Ang and Van Dyne 2008; Earley and Ang 2003), meaning that the level of interpersonal abilities that individuals possess within a culture is independent of the level of interpersonal abilities that those individuals possess across cultures (Earley and Ang 2003; Groves and Feyerherm 2011). Thus, in multicultural teams, in addition to EQ, individuals must also rely on cross-cultural abilities, like cultural intelligence (CQ), for effective interaction (Johnson et al. 2006; Thomas et al. 2008). CQ facilitates effectiveness in multicultural teams as individuals with higher CQ behave more effectively in culturally diverse situations (Earley and Ang 2003). CQ, defined as the ability of individuals to perform effectively in cross-cultural settings (Ang and Van Dyne 2008), has been shown as a good predictor of team performance (Groves and Feyerherm 2011; Presbitero 2016; Presbitero and Toledano 2018; Rockstuhl et al. 2015) and interpersonal process effectiveness (Presbitero 2021), like knowledge sharing (Ali et al. 2019; Chen and Lin 2013) in multicultural teams. Thus, it seems reasonable to suggest that not only cognitive ability influences team outputs, but also other types of intelligences may play a role in different types of teams. For example, multicultural teams' performance varies based on the team-level cultural diversity (Earley and Mosakowski 2000; Nakui et al. 2011), and team members' ability to effectively manage the emotions within their respective work teams (Davaei et al. 2022; Eberz et al. 2020). Therefore, team members' EQ and CQ could have critical impacts on multicultural teams' outcomes.

As the use of different types of work teams has gained substantial popularity in organizations (Davidaviciene et al. 2020; Kozlowski and Bell 2013; Neeley 2015), the selection of team members with essential competencies for successful teamwork has emerged as a top priority for firms. However, to the best of the authors' knowledge, a thorough analysis of the role of different types of intelligence in various team contexts has not been carried out so far. While an independent literature review is necessary to explain the current state and progress of the field to shed light on the gaps that exist and to chart the future trajectory of the field. Especially, current literature review articles on the role of intelligences in the workplace have mainly considered the impact of only one type of intelligence on a certain organizational process or outcome (e.g., Paiuc 2021; Reilly 2022), which makes it difficult to identify the major research themes and trends in the field and to identify noteworthy research gaps such as conflicting or inconclusive findings, emerging research theme, and underexplored research areas (Lim et al. 2022). Therefore, in the absence of a comprehensive and systematic literature review paper on this topic, this study seeks to present the prevailing state of research on the role of various types of intelligence in different processes and outcomes of various types of teams, with the following questions deciphering the scope of the study:

- RQ1: What types of intelligence have been examined in the teamwork setting?
- RQ2: What are the antecedents to, and outcomes of intelligences in a teamwork setting?
- RQ3: What are the processes by which intelligences affects team processes and outcomes?
- RQ4: How has the research on the role of intelligences in organizational teams evolved over the years and what are the recent research trends in this domain?
- RQ5: What are the gaps and areas for future research?

Thus, this study intends to structure existing research, identify its current trends, and provide an overview of recent research strands and topics on the role of intelligences in organizational teams to determine the research gaps and to guild future research directions. Analysis of the articles selected enables the identification of three different types of intelligence (EQ, CQ, and cognitive ability) as important input factors impacting different team processes and outcomes. The results reveal a greater focus on the study of EQ in face-to-face monocultural teams, while in recent years, there is an increasing interest in the research on the role of CQ in GVTs. The results of this systematic literature review contribute to the literature on (international) human resources management, specifically with regard to the role of intelligences in team member selection. The antecedents to, the outcomes of, and the processes by which intelligences affect team outcomes are discussed. More importantly, suggestions for future studies as a point of departure for broadening and deepening the literature on the role of intelligences in teamwork settings are provided.

## Methodology

This study applies a comprehensive, thorough, and concept-context hybrid systematic literature review (Tranfield et al. 2003) to analyze and structure the existing literature on the role of intelligences in organizational teams and to identify areas for further research. The selected method follows a transparent and reproducible methodology in searching, assesses the quality of the existing body of literature, and synthesizes them (Kraus et al. 2020). Therefore, it differs from the traditional narrative review, which uses unrepresentative samples and unsystematic procedures, thus often regarded to be ‘unscientific’ (Mulrow 1994; Oakley 2002). Moreover, a concept-context hybrid review can provide finer-grained insights and offer more detailed, nuanced, and specific information about a particular concept (e.g., intelligences) in a specific context (e.g., organizational teams) (Kraus et al. 2022a).

To analyze the literature, we used qualitative content (thematic) analysis. Content analysis enables scholars to analyze a small to medium corpus of articles by subjectively organizing the content into themes. (Kraus et al. 2022a). The qualitative content analysis enables the researchers to qualify and analyze the existence, meanings, and connections among certain words, themes, and/or concepts within particular qualitative data to make generalizable inferences (Elo and Kyngäs 2008). By using a qualitative content analysis in this study, we can gain a stronger grasp of the nature, evolution, and scope of the literature on the role of intelligences in organizational teams.

This study follows the steps laid out by Kumar et al. (2021) and Kushwaha et al. (2021) to structure the analysis. The review process is iterative, moving through three process steps: (1) planning the review, (2) conducting the review, and (3) reporting the review. The last step is presented in Sect. 3 (Results).

### Planning the review

Systematic literature reviews follow an inductive reasoning approach, where a specific set of criteria, like search database, search keywords, subject area, etc., is defined and applied to develop a corpus of scholarly documents, leading to a well-scoped, structured, integrated and interpreted/narrated review of the literature (Kraus et al. 2022a). For this study, we followed the criteria and data selection process as outlined by Kraus et al. (2022b). To identify relevant articles for this study, the online Web of Science (WoS) and EBSCO databases were utilized. WOS was chosen as it is one of the world's premier databases for published articles and citations and includes publications in top-tier journals and is most suited for literature review (Korom 2019). EBSCO was selected due to its variety of resources and its ability to search multiple databases, such as Emerald, the Sage, Blackwell, and Science Direct, simultaneously, hence it has great potential for a systematic review study (Bhimania et al. 2019).

We restricted the search only to English academic journal articles in the areas of business and management. While we acknowledge that intelligence has been a topic in multiple streams of literature, here, we only review publications in the areas of business and management to increase the relevancy of selected journals. To further restrict the search result to higher quality articles, i.e., applying a ‘quality threshold’ (see e.g., Bouncken et al. 2015), we only included the articles published in Chartered Association of Business Schools (ABS) ≥ 2-star journals.

### Conducting the review

**Fig. 1:**
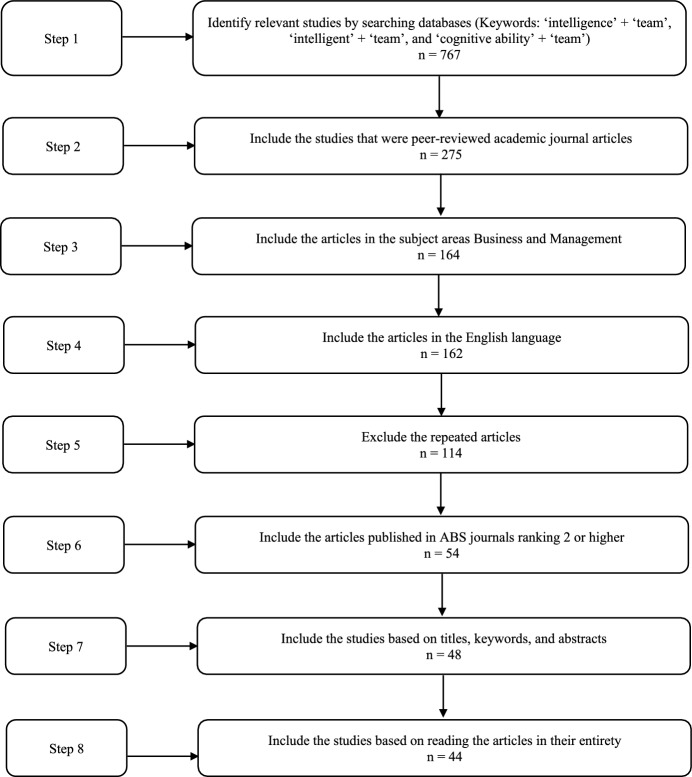
The data selection process

The searches were carried out using the terms ‘intelligence’ + ‘team’, ‘intelligent’ + ‘team’, and ‘cognitive ability’ + ‘team’ included in the article titles, which resulted in 767 articles. In the next step, the search was restricted only to academic journal articles, reducing the number of possible contributions to 275. In step three, the search was narrowed down further by focusing only on those contributions published in the areas of business and management. Thus, the number of contributions was reduced to 164. In the fourth step, the non-English articles were filtered out. This reduced the number of contributions to 162. In step five, duplications of articles were eliminated to avoid counting a paper twice in the analysis, which reduced the number of articles to 114. To increase the quality of data, in step six, only journals with a rating of ‘2’ and higher in the ABS ranking were included, which diminished the number of possible contributions to 54. To ensure the basic criteria of relevance and quality, in step seven, the titles, keywords, the abstracts of the remaining articles were screened, and the non-relevant articles were eliminated, reducing the number of contributions to 48. Finally, all 48 articles were read in their entirety and judged in terms of relevance to the role of intelligences in organizational teams. The final dataset that was analyzed included a total of 44 peer-reviewed academic journal articles published over the 23-year time period. The review procedure is summarized in Fig. 1.

The selected articles were published between 1999 and 2021 in 29 ABS journals, ranking two or higher, and were produced by 106 authors (115 author appearances), of which seven articles were published by a single author, and 37 articles were published as part of a co-authorship. Table 1 provides a descriptive summary of the articles included in the present study.

**Table 1:** Summary of articles resulting from the searches

Description	Results
Documents	44
Sources (journals)	29
Keywords plus (WOS)/Subject terms (EBSCO)	181
Author's keywords	105
Period	1999–2021
Average citations per document	101.57
Authors	106
Author appearances	115
Single-authored documents	7
Multi-authored documents	37
Documents per author	0.42
Authors per document	2.41

After determining all the studies that should be included, we categorized the covered articles based on the type of intelligence studied to reveal how each intelligence type affects various team processes and outcomes. In doing so, we looked for the type of intelligence in the title of the articles. Three different types of intelligence (three clusters) were identified, namely, emotional intelligence, cultural intelligence, and cognitive ability. The studies that examined the impacts of more than one intelligence on team processes and outcomes were categorized under the fourth cluster.

**Fig. 2:**
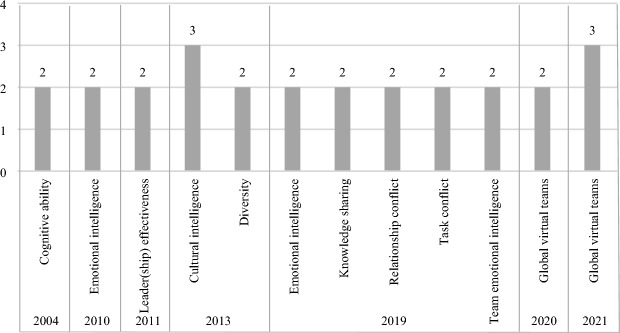
Overview of the most frequently used keywords

Furthermore, in order to learn more about the possible research trends over time, an analysis of the keywords frequently used in each year was performed. For a few articles that the keywords had not been specified by the authors, ‘Keywords Plus’ in the WOS database and ‘Subject Terms’ in the EBSCO database were used. This analysis was done for the years with keywords co-occurring in two or more articles. The results of the analysis are presented in Fig. 2. As seen, the keyword ‘cognitive ability’ was included in both articles studied in 2004. While, in 2010, ‘emotional intelligence’ appeared in both articles studied in this year. In 2011, the most frequently used keywords were ‘leader effectiveness’ and ‘leadership effectiveness’, included in both articles in this year. In 2013, the keywords ‘cultural intelligence’ (with three articles out of four total articles) and ‘diversity’ (with two articles out of four total articles) headed the list of the most frequently used keywords.

2019 was the year with the most publications on intelligences in teams in the covered studies. The keywords ‘emotional intelligence’, ‘knowledge sharing’, ‘relationship conflict’, ‘task conflict’, and ‘team emotional intelligence’ most frequently occurred in 2019 (with two articles each out of seven total articles). In 2020, the keyword ‘global virtual teams’ headed the list of the most frequently used keywords, with two articles out of three articles. The interest in research on GVTs could be due to the COVID-19 pandemic that has made remote work a reality for many organizations. This trend continued in 2021, with three articles out of four total articles, as the pandemic still had an effect on organizations and people's lives.

## Results (Reporting the review)

In this section, qualitative content analysis has been applied to analyze the literature on the role of intelligences in the organizational teams. This section categorizes the key findings of covered studies based on the four clusters identified in Sect. 2.2, i.e., emotional intelligence, cultural intelligence, cognitive ability, and multiple intelligences. In each section below, we discuss how each type of intelligence influences various organizational processes and outcomes.

### Emotional intelligence

The first cluster of articles, including 18 papers (40.9% of the total covered articles), discussed the role of EQ in team processes and outcomes. The majority of articles, 12 articles (66.67%), focused on either the direct or indirect relationship between EQ and performance in teams.

The results of content analysis show that team members' EQ directly improves individual performance in teams. Paik et al. (2019) investigated the relationship between EQ and individual performance in self-managing teams. The result indicates that team members' EQ is a robust and salient predictor of their performance in directing and coordinating collective efforts in self-managing teams where the task involves processing a heavy load of affective information. Moreover, the positive contribution of EQ on team members' performance is stronger for teams with higher diversity, larger sizes, and lower team EQ. Açikgöz and Latham (2020) studied the impact of perceived EQ on the adaptive performance of individuals working in new product development teams and found that perceived EQ is positively and significantly associated with adaptive performance. Zhao and Cai (2021), however, examined the indirect effect of team members' EQ on team performance. They discussed that one of the central mechanisms by which team members' EQ affects team performance is by influencing interpersonal interactions (Law et al. 2004), including leader-member exchange (LMX) and team-member exchange (TMX). They found that a team member's EQ has a positive impact on TMX, and TMX, in turn, positively influences team performance. Moreover, they proposed that different dimensions of EQ have varying effects on TMX, with ‘regulation of emotion’ having the strongest effect on TMX. They also confirmed that EQ-LMX relation is positive at the individual level, and EQ variety within a team is positively associated with LMX differentiation at the team level. They suggested that when the leader differentiates team members based on their EQ diversity and develops high-quality exchange with high-EQ members, high EQ is more likely to help members develop high-quality TMX.

The direct impact of team-level EQ on team performance has also been studied in a number of articles included in this literature review. Macht et al. (2019) tested the relationships between team EQ and team performance with respect to five operationalization methods: mean, standard deviation, maximum, minimum, and proportion. They suggested that EQ's mean, maximum, and minimum aggregation methods should be considered when looking at team performance. They also found that the three main EQ sub-scales of intrapersonal skills, stress management, and adaptability are significant predictors of team performance. Jordan et al. (2002) suggested that team-level EQ predicts team performance. High EQ teams operated at high levels of performance throughout the study period. On the other hand, low EQ teams initially performed at a low level but raised their performance to match that of teams with high EQ over time. Günsel and Açikgöz (2013) explored the influence of team flexibility and team EQ on the performance of the software development teams. They verified that the diversity of software development teams (one of the team flexibility dimensions) improves the team's overall EQ. They also demonstrated that emotional recognition (one of the EQ dimensions) affects the speed to market and functionality of the new software products (two dimensions of performance). Jamshed and Majeed (2019) also found that team EQ is a predictor of performance; in fact, they proposed that team EQ mediates the relationship between team culture and team performance. The team EQ has also been suggested to influence the team's output and functioning. Similarly, Lee and Wong (2017) found that team EQ is positively related to team performance.

Team EQ can also indirectly affect team performance via a moderation effect. Kaufmann and Wagner (2017) investigated the potentially harmful effects of affective trait diversity on sourcing team performance and how such adverse effects might be mitigated through the team EQ. They found that the sourcing team's EQ positively moderates the link between affective diversity and team cohesion and subsequently has a positive influence on sourcing team performance. Thus, the higher the team EQ, the weaker the negative affective diversity-team cohesion relationship. Lee and Wong (2017) examined the moderating effect of a team EQ on the relationship between team process (task conflict and relationship conflict) and team effectiveness (team performance, cohesion, and innovation). The results demonstrate that team EQ reduces the adverse effects of task conflict on team performance, team cohesion, and innovation.

The results of the content analysis also indicate that the team EQ-team performance relationship can be mediated by various team processes, such as the team's shared mental model, intra-team trust, and social network structure. Xiang et al. (2016) proposed that it is helpful to improve the team performance of information system development teams by choosing the team members based on EQ but building a higher level of the team's shared mental model could make such impact even better. They also suggested that the awareness of own emotions and management of own emotions could improve the task-related shared mental model (shared vision of task) and member-related shared mental model (common sense of members) in information system development teams. While management of others' emotions could only influence the member-related shared mental model. Chang et al. (2012) explained the pathways by which EQ at the team level affects team performance by investigating the intervening dynamics. They proposed that team EQ is a significant predictor of intra-team trust, which mediates the link between a team EQ and team performance. In another study, Zhang et al. (2019) investigated the role of social network structure (i.e., friendship network density) as a mediating factor linking team EQ with team task performance and how this relationship is influenced by the intra-team trust. The results verify the positive team EQ-team performance relation. In addition, they found that teams with higher average EQ exhibited a higher density of friendship networks and better team task performance when working under greater intra-team trust conditions. Therefore, intra-team trust can have either a mediation (Chang et al. 2012) or moderation (Zhang et al. 2019) effect on the team EQ-team performance relationship.

Team trust has also been suggested to mediate the relationship between the team EQ and other team processes and outcomes. For example, Barczak et al. (2010) examined the impact of a team EQ on team trust and the effect of team trust on the team's collaborative culture and creativity. Their results show that emotionally intelligent teams create both cognitive and affective team trust. More specifically, awareness of own emotions and management of others' emotions are positively and significantly related to affective trust. While management of own emotions and management of others' emotions are significant in explaining cognitive trust among team members. The team trust, in turn, helps build a collaborative culture leading to higher levels of team creativity.

The impact of team EQ on intragroup conflicts has been investigated in two studies. Lee and Wong (2017) examined the effects of a team EQ on team process (task conflict and relationship conflict) and team effectiveness (team performance, cohesion, and innovation). The results show that team EQ is negatively associated with task conflict and relationship conflict and positively related to team performance, team cohesion, and innovation. A team EQ also reduces the adverse effects of relationship conflict on team cohesion. Moreover, as task conflict results in a relationship conflict, it has been argued that task conflict has a detrimental effect on team effectiveness. Thus, they investigated the team EQ as a contextual factor that decouples the overlooked link between task conflict and relationship conflict. The results show that the team EQ plays a significant role in decoupling task conflict and relationship conflict. Ayoko et al. (2008) investigated the direct effects of team EQ climate (team empathic concern, team emotion management, and team conflict management norms) on conflict types (task and relationship) and conflict features (intensity and duration). They further examined the moderation effect of the different team EQ climate dimensions on the link between both conflict types and conflict features and team members' reactions to conflict (productive and destructive). The result demonstrates that lower levels of different dimensions of team EQ climate are related to increased task conflict, relationship conflict, and conflict intensity. Furthermore, the three dimensions of team EQ climate are positively associated with prolonged conflict duration. Meaning that too much empathic concern, emotion management, and team conflict management norms can be counterproductive; thus, to reduce chronic conflict in the team, managers may need to achieve a balanced amount of team EQ climate. Moreover, they found that teams with task conflict but with lower levels of team conflict management norms reported destructive reactions to conflict, suggesting that when task conflict is managed through conflict management norms, destructive reactions to a conflict may be lessened. Team conflict management norms also moderated conflict intensity for destructive reactions to conflict. Meaning that teams that experience conflict intensity but with fewer team conflict management norms also experience destructive reactions to conflict.

Only two studies examined the impact of a team leader's EQ on various team outcomes. Hur et al. (2011) examined whether transformational leadership mediates the link between the EQ of team leaders and three outcomes as perceived by followers: leader effectiveness, team effectiveness, and service climate. They found that transformational leadership mediates the relationships between a leader's EQ and leader effectiveness, as well as between a leader's EQ and service climate, although not between a leader's EQ and team effectiveness. Chang et al. (2012) demonstrated that a leader's EQ not only directly influences intra-team trust and team performance but also moderates the effect of the team EQ on team emergent states and outcomes. They also suggested that the team EQ and leader EQ have a compensatory relationship in predicting team performance, such that either a high team EQ or a high leader EQ, not necessarily both, is sufficient to explain a high level of team performance. This pattern is particularly strong with the emotion appraisal and social skills dimensions of EQ.

Two articles included in our covered studies have contributed to the literature by developing an EQ measurement model. Jordan et al. (2002) developed and validated a new EQ scale, the Workgroup Emotional Intelligence Profile, Version 3 (WEIP-3), to measure the EQ of individuals working in teams. They further applied the scale in a study of the link between team EQ and team performance. Jordan and Lawrence (2009) further outlined a theoretical model for measuring EQ abilities vital during the interaction of teamwork. They used this model to test a short version (16 items) of the self-report WEIP (Jordan et al. 2002), including four distinct constructs: awareness of own emotions; management of own emotions; awareness of others' emotions; and management of others' emotions. The results provide evidence of the reliability and construct validity of the WEIP-S.

Lastly, Cole et al. (2016) investigated the role of EQ and SOAR (strengths, opportunities, aspirations, and results) as strategies to support the collaborative process. The results suggest that increases in EQ among team members improve team member collaboration. EQ also appears to be a stronger positive predictor of collaboration in team members working in face-to-face teams than in virtual teams. Furthermore, the results of the mediation analyses suggest that as strengths and results increase and aspirations decrease, EQ has a significant positive effect on collaboration. Clarke (2010a) examined the impacts of attending a one-day EQ training program (EQ self-awareness) followed by participating in team-based learning on ability-based measures of EQ. The result indicates that the EQ training alone does not affect the development of EQ abilities; however, when followed by participating in intense team-based learning, the EQ ability to use emotions to facilitate thinking, relating to individuals becoming more conscious of their emotions and using these in their decision making, could be developed. However, the intensity of an individual's participation in team-based learning appears to play a critical role. Regarding the other EQ abilities to improve, far longer or more intense periods of team-based training have been suggested.

Table 2 presents the EQ measures, sample characteristics, type of teams, and key findings of covered articles in this cluster.

**Table 2:** EQ measure, sample characteristic, type of team, and key findings

Authors	EQ measure	Team type	Sample	Key findings
Açikgöz and Latham (2020)	WLEIS	Face-to-face teams	Employees	Perceived EQ is positively and significantly associated with adaptive performance in new product development teams
Ayoko et al. (2008)	Adapted from WEIP and Jehn (1997)	Face-to-face teams	Employees	Different dimensions of team EQ climate (team empathic concern, team emotion management, and team conflict management norms) are negatively related to task conflict, relationship conflict, and conflict intensity and positively associated with prolonged conflict duration Teams with task conflict but with lower levels of team conflict management norms (one of the team EQ climate dimensions) experience destructive reactions to conflict Teams that experience conflict intensity but with fewer team conflict management norms also experience destructive reactions to conflict
Barczak et al. (2010)	WEIP-S	Face-to-face teams	Students	Awareness of own emotions and management of others' emotions are positively and significantly related to affective trust Management of own emotions and management of others' emotions are significant in explaining cognitive trust among team members Team trust mediates the relationship between the team EQ and the team's collaborative culture, leading to higher levels of team creativity
Chang et al. (2012)	EIS	Face-to-face teams	Employees	Team EQ is a significant predictor of intra-team trust, which mediates the link between a team EQ and team performance A leader's EQ not only directly influences intra-team trust and team performance but also moderates the effect of the team EQ on team emergent states and outcomes Team EQ and leader EQ have a compensatory relationship in predicting team performance
Clarke (2010a)	MSCEIT V2.0	Multicultural teams	Students	When EQ training follows by participating in intense team-based learning, the EQ ability to use emotions to facilitate thinking can be developed
Cole et al. (2016)	WEIP-S	Both face-to-face and virtual teams	Employees	EQ is positively related to team member collaboration EQ is a stronger positive predictor of collaboration in team members working in face-to-face teams than in virtual teams
Günsel and Açikgöz (2013)	WLEIS	Face-to-face teams	Employees	Diversity of software development teams improves the team overall EQ Emotional recognition of team members affects the speed to market and functionality of the new software products
Hur et al. (2011)	WLEIS	Face-to-face teams	Employees	Transformational leadership mediates the relationships between a leader EQ and leader effectiveness, as well as between a leader EQ and service climate
Jamshed and Majeed (2019)	WEIP-S	Face-to-face teams	Employees	Team EQ mediates the relationship between team culture and team performance Team EQ influences the team's output and functioning
Jordan and Lawrence (2009)	WEIP-S	Face-to-face teams	Employees	The evidence of the reliability and construct validity of the WEIP-S is presented
Jordan et al. (2002)	WEIP-3	Face-to-face teams	Students	Team-level EQ predicts team performance Teams with high EQ operate at a high level of performance throughout the teamwork. On the other hand, low EQ teams initially perform at a low level but will raise their performance to match that of teams with high EQ over time
Kaufmann and Wagner (2017)	WLEIS and WEIP-S	Face-to-face teams	Employees	Team EQ positively moderates the link between affective diversity and team cohesion and subsequently has a positive influence on team performance
Lee and Wong (2017)	WEIP-S	Face-to-face teams	Employees	Team EQ is negatively associated with task conflict and relationship conflict and positively related to team performance, team cohesion, and innovation Team EQ reduces the adverse effects of task conflict on team effectiveness (team performance, team cohesion, and innovation) Team EQ reduces the adverse effects of relationship conflict on team cohesion The team EQ plays a significant role in decoupling the link between task conflict and relationship conflict
Macht et al. (2019)	EQ-i	Face-to-face teams	Students	EQ's mean, maximum, and minimum aggregation method should be considered when looking at team performance EQ sub-scales of intrapersonal skills, stress management, and adaptability are significant predictors of team performance
Paik et al. (2019)	MSCEIT V2.0	Face-to-face teams	Students	Team members' EQ improves the performance of individuals in self-managing teams. This relationship is stronger for teams with higher diversity, larger sizes, and lower team EQ
Xiang et al. (2016)	WEIP-S	Face-to-face teams	Employees	Team shared mental model mediates the relationship between EQ and performance in information system development teams The awareness of own emotions and management of own emotions could improve the task-related shared mental model and member-related shared mental model in information system development teams. While management of others' emotions only influences the member-related shared mental model in such teams
Zhang et al. (2019)	WLEIS	Face-to-face teams	Students	Team EQ has a positive relationship with team performance Teams with higher average EQ have a higher density of friendship networks and better team task performance when working under greater intra-team trust conditions
Zhao and Cai (2021)	WLEIS	Face-to-face teams	Employees	Team members' EQ has a positive impact on TMX, and TMX, in turn, positively influences performance EQ-LMX relation is positive at the individual level and EQ variety within a team is positively associated with LMX differentiation at the team level When the leader differentiates team members based on their EQ diversity and develops high-quality exchange with high-EQ members, high EQ is more likely to help members develop high-quality TMX

### Cultural intelligence

Papers assigned to this cluster address the importance of CQ in multicultural teams. This cluster includes 15 articles (34.09% of the total covered articles). Almost half of the studies in this cluster, seven articles (46.67%), examined the direct or indirect relationships between CQ and performance in multicultural teams.

The results of the content analysis indicate that team members' CQ has a direct impact on individual performance in multicultural teams. Hu et al. (2019) examined the impact of individual-level CQ on employees' creative performance in inter-organizational teams. The results reveal that CQ is positively related to employees' creative performance, and this positive relationship is strengthened in the context of high relationship conflict and low task conflict. Presbitero and Toledano (2018) examined the development of global team members' CQ following cross-cultural training and its effects on individual-level task performance. The results suggest that cross-cultural training increases team members' CQ, and improved CQ boosts individual-level task performance. Moreover, contact intensity has a moderating effect on the relationship between improved CQ and individual-level task performance.

At the team level, Richter et al. (2021) examined the impact of CQ (minimum and maximum CQ of members, team's average CQ, and leader's CQ) on social integration and team performance in GVTs. The results reveal that the team's average motivational CQ is a necessary condition for high levels of GVTs' social integration and performance. Furthermore, an increase in the team's average motivational CQ will improve the social integration and performance of GVTs. Similarly, an increase in the motivational CQ of either the team member with the highest CQ or the team member with the lowest CQ improves the team's social integration*.*

The results also show that CQ can serve as a moderator, which changes the nature of the relationship between a predictor and performance in multicultural teams. Moon (2013) examined the relative performance changes of multicultural teams over time, as well as the relationship between a team's overall CQ and performance in such teams. The results reveal that the degree of cultural diversity influences team performance in multicultural teams over time and that teams with higher CQ perform better at the initial stage of team interactions than those with lower CQ. Furthermore, teams with higher CQ not only reduce the negative relationship between cultural diversity and initial team performance but also improve their performance more quickly than teams with lower CQ. Presbitero (2020a) developed and tested a moderated-moderation model involving a member's task performance in GVTs. The results demonstrate that team members' perceived cultural dissimilarity is negatively and significantly related to their task performance. Furthermore, the CQ of team members moderates the relationship between perceived cultural dissimilarity and individual task performance. This moderation effect is also moderated by the team leader's CQ. In another study, Presbitero (2020b) suggested that foreign language use in GVTs influences individual task performance. He further proposed that CQ serves as a condition for achieving a high level of individual task performance despite the presence of a high level of foreign language anxiety, meaning that a high level of CQ decreases the negative effect of foreign language anxiety on the individual task performance of GVT members. Henderson et al. (2018) tested how CQ moderates the way team members' role clarity and interpersonal trust in global project teams indirectly affect the impact of communication norms on their performance and satisfaction. The results of a moderated-mediation analysis demonstrate that motivation CQ significantly moderates team members' alignment of communication norms and role clarity. Meaning that when team members' motivation CQ is high, their role clarity improves with higher levels of alignment in their communication norms, thus indirectly impacting their project satisfaction and performance. Moreover, the results of post hoc analysis revealed that motivation CQ significantly mediates the impact of communication norms on team members' satisfaction.

The role of the leader CQ in the team's processes and outcomes has also been investigated in the covered studies. According to Presbitero's (2020a) moderated-moderation model, the CQ of team members moderates the relationship between perceived cultural dissimilarity and task performance. The magnitude of the moderation is contingent upon the team leader's CQ level. In other words, a higher level of a team leader's CQ yields higher moderation effects. Richter et al. (2021) proposed that improving a leader's motivational CQ will increase the social integration and performance of GVTs. Furthermore, the motivational CQ of the team leader is a necessary condition for the social integration of GVTs, but not for the team's performance*.* Presbitero and Teng-Calleja (2019) proposed that through the mechanisms of social learning and role modeling, perceived ethical leadership (as perceived by team members) is positively and significantly related to the ethical behavior of individual members of global teams. Furthermore, the perceived CQ of leaders, which consists of perceptions of members regarding the leader's cultural knowledge and skills on how to act ethically in different cultural contexts, serves as a moderator in strengthening the relationship between perceived ethical leadership and individual members' display of ethical behavior.

Three studies included in our literature review discussed CQ as a key driver of Knowledge sharing in multicultural teams. Chen and Lin (2013) proposed that CQ is a key driver of knowledge sharing in multicultural teams. Their study shows that knowledge sharing is positively and directly influenced by metacognitive, cognitive, and motivational CQ. Moreover, metacognitive CQ and behavioral CQ have indirect and positive effects on knowledge sharing through the mediation of perceived team efficacy. Ali et al. (2019) examined the moderation effect of expatriate employees' CQ on the relationship between expatriate employees' knowledge sharing with home country national employees and individual and team creativity. The result suggests that the relationship between expatriate employees' knowledge sharing with home country national employees and creativity is stronger when CQ is high and weaker when CQ is low. In a similar vein, Bogilović et al. (2017) argued that employees hide knowledge from culturally diverse colleagues, which in turn, will inhibit individual creativity. They also proposed that the relationship between individual knowledge hiding, and individual creativity is less harmful when moderated by CQ.

Erez et al. (2013) and Presbitero and Toledano (2018) argued the possibility of improving individuals' CQ through training and learning opportunities. Presbitero and Toledano (2018) suggested that cross-cultural training increases team members' CQ. Erez et al. (2013) investigated the possibility of enhancing team members' CQ and global identity through their participation in a virtual multicultural team project that was designed according to the construction-group model of experiential learning. The results show a significant increase in CQ and global identity while the project was conducted. Moreover, the effect of the multicultural team project on team members' CQ and global identity remains stable beyond the project period. The results also highlight the importance of trust in virtual multicultural teams, as team-level trust contributes to the increase in CQ and global identity. Furthermore, team-level trust fully moderates the change over time in global identity and marginally moderates the change over time in the overall measure of CQ.

The impact of CQ on interpersonal process effectiveness and employee engagement has been examined in two studies. Presbitero (2021) examined how CQ influences the interpersonal process effectiveness of GVT members. The results show that a high level of CQ relates to high levels of effectiveness in GVT members' display of synergy and direction effectiveness. Moreover, the communication accommodation of a GVT member is impacted by CQ, which consequently affects both effectiveness of interpersonal processes of synergy and direction. Shaik et al. (2021) performed an ethnographic inquiry to examine the linkages between CQ and employee engagement based on work and non-work identities. The results demonstrate that the inclusionary pressures of non-work identities (national culture) are high in the GVTs context. However, preferences of team members (alignment or misalignment) initiate either gain cycles or loss cycles, thus affecting employee engagement levels. Furthermore, they found that improving team members' CQ may dynamically change their preferences from misalignment toward alignment in GVTs. The relationship between CQ and employee engagement is mediated by trust among team members in GVTs.

In the last study of this cluster, Janssens and Brett (2006) developed a new, culturally intelligent model of collaboration for global teams to use during their face-to-face meetings. This fusion model provides guidelines that are intended to reduce process losses and enhance the likelihood of such teams making creatively realistic decisions. They proposed that the fusion model is more culturally intelligent than other models of team collaboration previously discussed in the literature, like the dominant coalition or the integration/identity models (Canney Davison 1996; Canney Davison and Ward 1999). In this model, a structural intervention, fusion, has been proposed that has the CQ, or the ability to transform the processes of the group, built into its principles. They suggested that team members become more culturally intelligent, using the fusion model of collaboration in global teams.

Table 3 demonstrates the CQ measures, sample characteristics, type of teams, and key findings of articles in this cluster.

**Table 3:** CQ measure, sample characteristic, type of team, and key findings

Authors	Measure	Team type	Sample	Key findings
Ali et al. (2019)	Ang and Van Dyne (2008)	Teams including expatriates and host country national employees	Employees	The relationship between expatriate employees' knowledge sharing with home country national employees and creativity is stronger when CQ is high
Bogilović et al. (2017)	Ang and Van Dyne (2008)	Multicultural teams	Study 1: Employees Study 2: Students	The negative relationship between employees' knowledge hiding from culturally diverse colleagues and individual creativity is less harmful when moderated by CQ
Chen and Lin (2013)	Ang et al. (2007)	Multicultural teams	Employees	Knowledge sharing is positively and directly influenced by metacognitive, cognitive, and motivational CQ Metacognitive CQ and behavioral CQ have indirect and positive effects on knowledge sharing through the mediation of perceived team efficacy
Erez et al. (2013)	Ang et al. (2006, 2007)	GVTs	Students	Participating in a virtual multicultural team project increases team members' CQ and global identity during the project. The effect of the multicultural team project on team members' CQ and global identity remains stable beyond the project period Team-level trust contributes to the increase in CQ and global identity Team-level trust marginally moderates the change over time in the overall measure of CQ
Henderson et al. (2018)	Van Dyne et al. (2008)	GVTs	Employees	When team members' motivation CQ is high, their role clarity improves with higher levels of alignment in their communication norms, thus indirectly impacting their project satisfaction and performance Motivation CQ significantly mediates the impact of communication norms on team members' satisfaction
Hu et al. (2019)	Ang et al. (2007)	Inter-organizational teams	Employees	Individual-level CQ is positively related to employees' creative performance, and this positive relationship is strengthened in the context of high relationship conflict and low task conflict
Janssens and Brett (2006)	–	Global face-to-face teams	–	A new, culturally intelligent model of collaboration for global teams is proposed. In this model, a structural intervention, fusion, is proposed that has the CQ, or the ability to transform the processes of the group, built into its principles Team members become more culturally intelligent, using the fusion model of collaboration in global teams
Moon (2013)	Ang et al. (2004)	Multicultural teams	Students	Teams with higher CQ perform better at the initial stage of team interactions than those with lower CQ Teams with higher CQ not only reduce the negative relationship between cultural diversity and initial team performance but also improve their performance more quickly than teams with lower CQ
Presbitero and Teng-Calleja (2019)	Thomas et al. (2015)	Global teams	Employees	Perceived ethical leadership (as perceived by team members) is positively and significantly related to the ethical behavior of individual members of global teams and the perceived CQ of leaders serves as a moderator in strengthening this relationship
Presbitero and Toledano (2018)	Ang et al. (2007)	Global teams	Employees	Cross-cultural training increases team members' CQ, and improved CQ boosts individual-level task performance Contact intensity has a moderating effect on the relationship between improved CQ and individual-level task performance
Presbitero (2020a)	Ang and Van Dyne's (2008) (short version)	GVTs	Employees	CQ of team members moderates the negative relationship between perceived cultural dissimilarity and task performance. The magnitude of the moderation is contingent upon the team leader CQ level
Presbitero (2020b)	Thomas et al. (2015)	GVTs	Employees	A high level of CQ decreases the negative effect of foreign language anxiety on the individual task performance of GVT members
Presbitero (2021)	Ang and Van Dyne (2015)	GVTs	Employees	High level of CQ relates to high levels of effectiveness in GVT members' display of synergy and direction effectiveness The communication accommodation of a GVT member is impacted by CQ, which consequently affects both effectiveness of interpersonal processes of synergy and direction
Richter et al. (2021)	Ang and Van Dyne (2008)	GVTs	Students	The team's average motivational CQ is a necessary condition for high levels of GVTs' social integration and performance An increase in the team's average and/or leader's motivational CQ will improve the social integration and performance of GVTs An increase in the motivational CQ of either the team member with the highest CQ or the team member with the lowest CQ improves the team's social integration The motivational CQ of the team leader is a necessary condition for the social integration of GVTs, but not for the team performance
Shaik et al. (2021)	–	GVTs	Employees	The relationship between CQ and employee engagement is mediated by trust among team members in GVTs

### Cognitive ability

This cluster contains five papers (11.36% of total papers) that aim to underscore the impact of cognitive ability on performance in organizational teams.

Neuman and Wright (1999) evaluated the effectiveness of using cognitive ability to predict performance at both individual and team levels. They found that team-level cognitive ability and team members' cognitive ability, as measured by the lowest scoring member of teams performing conjunctive tasks, respectively, predict team performance and individual performance. They also found that personality measures predict performance in teams beyond cognitive ability at both individual and team levels. Devine and Philips (2001) reported the results of several meta-analyses examining the relationship between team-level cognitive ability and team performance using four operational definitions of cognitive ability within teams (i.e., mean score, highest member score, lowest member score, and standard deviation of scores). The results reveal that the standard deviation of member cognitive ability scores generally appears to be unrelated to team performance, whereas the mean, highest member score, and lowest member score operational definitions of team-level cognitive ability have positive relationships with measures of team performance. Moreover, the strength of the positive relationship is moderated by other variables for all three of these indices. The results also show that the mean cognitive ability is a much weaker predictor of team performance in organizations than in the lab.

LePine (2003) suggested that teams including members with higher cognitive ability tend to be effective at adapting their role structure (i.e., the effectiveness with which teams adapt their role structure when faced with an unforeseen change in their task context). Also, these teams tend to make more accurate decisions and perform better than teams with members who scored low on cognitive ability. Later, he extended this study and found that such teams have a higher likelihood of adaptation by the time the deterioration stabilized (LePine 2005). Summers et al. (2012) studied the conditions under which team member change results in team coordination flux and consequently affects team performance. Results reveal that team member change leads to high levels of flux in coordination when either a member changes to a more strategically core role or there is low information transfer during the change. Moreover, in the case of the team member change to strategic core roles, the impact of team member change on coordination flux is stronger when the new member's relative cognitive ability is low. Also, the effect of the interaction term on task performance, as mediated by the flux in coordination, is weaker under the condition of high new member relative cognitive ability.

Table 4 presents the measures used to assess cognitive ability, sample characteristics, type of teams, and key findings of covered articles in this cluster.

**Table 4:** Cognitive ability measure, sample characteristic, type of team, and key findings

Authors	Measure	Team type	Sample	Key finding
Devine and Philips (2001)	(a) scores from an established measure of cognitive ability (e.g., Wonderlic Personnel Test 1992) (b) General aptitude (e.g., American College Test Verbal, Scholastic Assessment Test Quantitative) (c) Multi-aptitude test scores (e.g., composite scores on the General Aptitude Test Battery or Differential Aptitude Test)	–	–	Team-level cognitive ability, as measured by mean, highest member score, and lowest member score, have positive relationships with measures of team performance, and the strength of the positive relationship is moderated by other variables
LePine (2003)	Wonderlic Personnel Test (Form IV; Wonderlic and Associates 1992)	Virtual teams	Students	Teams, including members with higher cognitive ability, tend to be effective at adapting their role structure, which in turn, tend to make more accurate decisions and perform better
LePine (2005)	Wonderlic Personnel Test (Form IV; Wonderlic and Associates 1992)	Virtual teams	Students	After an unexpected change in the task, there will be a positive relationship between the level of team members' cognitive ability and role structure adaptation
Neuman and Wright (1999)	Thurstone Test of Mental Alertness (TMA; SRA 1988)	Face-to-face teams	Employees	Team-level cognitive ability and team members' cognitive ability, respectively, predict team performance and individual performance
Summers et al. (2012)	Wonderlic Personnel Test (Wonderlic and Associates 1983)	Face-to-face teams	Students	The impact of team member change to a more strategically core role on coordination flux is stronger when a new member's relative cognitive ability is low The indirect effect of team member change on task performance, as mediated by the flux in coordination, is weaker under the condition of high new member relative cognitive ability

### Multiple intelligences

This cluster which includes six articles (13.64% of total articles), encompasses contributions to research on teams that highlight the importance of multiple intelligences of team members. These studies underscore the interactive relations of more than one intelligence and their impacts on team processes and outputs.

In a general review paper, Green et al. (2005) suggested how team members' different degrees of multiple intelligences proposed by Gardner may be used to enhance their contribution to the team, leading to improved team productivity.

EQ and cognitive ability have been examined at the same time in three studies. Wolff et al. (2002) introduced and tested a model of the KSAs that predicts leadership emergence in self-managing teams. Based on this model, they highlighted the relevance of EQ and cognitive skills to the exhibition of the behaviors that predict the emergence of an informal leader. The findings support the basic premise of the model that empathy (an aspect of EQ) enables the cognitive skills of pattern recognition (the ability to synthesize information and identify patterns in a collection of unorganized information) and perspective taking (analyzing, discerning, and considering the merits of another's point of view (Boland and Tenkasi 1995)), which form the foundation for the leadership behaviors used by emergent leaders. The results also reveal that the cognitive skill of perspective-taking is directly, and the cognitive skill of pattern recognition is indirectly related to being chosen as an informal leader in self-managing teams. Offermann et al. (2004) investigated the impacts of emotional competence and cognitive ability on individual and team performance, team-member attitudes, and leadership perceptions. They suggested that although both cognitive ability and emotional competence predict performance, cognitive ability accounts for more variance in individual tasks. In comparison, emotional competence accounts for more variance in team performance and attitudes. They also proved that emotional competence is positively associated with attitudes toward one's team. Furthermore, individuals who score higher in emotional competence are more likely to be identified as team leaders and have better leader effectiveness. Sue-Chan and Latham (2004) examined whether cognitive ability and EQ explain the predictive validity of the situational interview. They found that both the situational interview and cognitive ability have predictive validity for the academic performance of managers and professionals in an executive MBA course. Moreover, the results revealed that EQ not only correlates with the situational interview, but it also completely mediates the relationship between the situational interview and team-playing behavior.

Groves and Feyerherm (2011) compared the effects of a leader's CQ with a leader's EQ on the performance in multicultural teams. In this study, they examined the relationship between a leader's CQ and followers' perceptions of both leader performance and team performance in multicultural teams. They found that leaders with higher CQ demonstrate greater team performance and leader performance in multicultural teams than in culturally homogeneous ones. Furthermore, a leader's CQ predicts follower perceptions of both leader performance and team performance in multicultural teams beyond the effects of a leader's EQ.

Boyatzis et al. (2017) examined the degree to which emotional and social intelligence might make a difference in the effectiveness of wildland fire incident commanders (the leaders of incident management teams). The results of 15 incident commanders' interviews indicate that five competencies of emotional self-control, coach and mentor, adaptability, empathy, and inspirational leadership were found to distinguish outstanding performers from average performer incident commanders. While achievement orientation, organizational awareness, influence, conflict management, and teamwork appeared as threshold competencies, being necessary competencies for achieving average performance but not sufficient alone to enable outstanding performance. Thus, these results suggest that social intelligence and EQ are important predictors of success in the incident commanders' role.

Table 5 represents intelligences' types and measures, sample characteristics, type of teams, and the key findings of articles studied in this cluster.

**Table 5:** Intelligences' type and measure, sample characteristic, type of team, and key findings

Authors	Intelligence type	Measure	Team type	Sample	Key findings
Boyatzis et al. (2017)	Social intelligence	A behavioral approach to ESI	Face-to-face teams	Employees	Social intelligence and EQ are important predictors of success in the incident commanders' role
	EQ	A behavioral approach to ESI			
Green et al. (2005)	Multiple intelligences	–	–	–	Team members' different degrees of multiple intelligences can be used to enhance their contribution to the team, leading to improved team productivity
Groves and Feyerherm (2011)	CQ	Ang et al. (2007)	Multicultural teams	Students	Leaders with higher CQ demonstrate greater team performance and leader performance in multicultural teams than in culturally homogeneous ones Leader CQ predicts follower perceptions of both leader performance and team performance in multicultural teams beyond the effects of a leader EQ
	EQ	WLEIS			
Offermann et al. (2004)	Cognitive ability	SAT Score	Face-to-face teams	Students	Both cognitive ability and emotional competence predict performance Cognitive ability accounts for more variance in individual tasks, while emotional competence accounts for more variance in team performance and attitudes Emotional competence is positively associated with attitudes toward one's team Individuals who score higher in emotional competence are more likely to be identified as team leaders and have better leader effectiveness
	Emotional competence	ECI-U (Boyatzis and Goleman 2002)			
Sue-Chan and Latham (2004)	Cognitive ability	Wonderlic Personnel Test and Scholastic Level Exam: User's Manual (2000)	Face-to-face teams	Students	Cognitive ability has predictive validity for academic performance EQ completely mediates the relationship between the situational interview and team-playing behavior
	EQ	WEIP-5			
Wolff et al. (2002)	Cognitive skills	Adapted from Boyatzis (1995)	Multicultural self-managing teams	Students	Empathy (an aspect of EQ) enables the cognitive skills which form the foundation for the leadership behaviors used by emergent leaders
	EQ	Adapted from Boyatzis (1995)			

## Discussion and conclusion

In today's dynamic, diverse, and ever-changing environment, organizations have increasingly turned to the use of work teams for the attainment of significant organizational goals or tasks (Alper et al. 2000; Martin 2001). The findings presented in this study reveal that different levels of intelligence that individuals bring to a team environment can provide additional value to the team's processes and effectiveness.

The majority of studies in our covered articles are empirical quantitative papers, in which an intelligence type (EQ, CQ, and cognitive ability) serves as a dependent variable, mediator/moderator, or independent variable. The content analysis of the literature review result shows that the relation of EQ with various team processes and outcomes has been mostly investigated at the team level (Ayoko et al. 2008; Barczak et al. 2010; Chang et al. 2012; Günsel and Açikgöz 2013; Hur et al. 2011; Jamshed and Majeed 2019; Jordan et al. 2002; Kaufmann and Wagner 2017; Lee and Wong 2017; Macht et al. 2019; Xiang et al. 2016; Zhang et al. 2019), while only three studies are at the individual level (Açikgöz and Latham 2020; Clarke 2010a; Cole et al. 2016), and two studies have the multilevel structure (Paik et al. 2019; Zhao and Cai 2021). The results indicate that team EQ positively improves team performance (Günsel and Açikgöz 2013; Macht et al. 2019; Jordan et al. 2002; Jamshed and Majeed 2019; Lee and Wong 2017; Zhang et al. 2019), team output and functioning (Jamshed and Majeed 2019), team's shared mental model (Xiang et al. 2016), team trust (Barczak et al. 2010; Chang et al. 2012), social network structure (Zhang et al. 2019), conflict duration (Ayoko et al. 2008), team cohesion (Lee and Wong 2017), and innovation (Lee and Wong 2017), and negatively affects the occurrence of intragroup conflicts (Ayoko et al. 2008; Lee and Wong 2017) and the intensity of intragroup conflicts (Ayoko et al. 2008). Also, a team's EQ indirectly influences team performance via a moderation effect (Kaufmann and Wagner 2017; Lee and Wong 2017). Team EQ-team performance relationship can also be mediated by the team's shared mental model (Xiang et al. 2016), intra-team trust (Chang et al. 2012), and social network structure (Zhang et al. 2019). Moreover, team EQ reduces the adverse effects of intragroup conflicts on team effectiveness. It also plays a significant role in decoupling the link between task conflict and relationship conflict (Lee and Wong 2017). A few studies have examined the direct effects of individual-level EQ on different team processes and outcomes. The results show that team members' EQ directly improves individual performance (Paik et al. 2019; Açikgöz and Latham 2020), team member collaboration (Cole et al. 2016), and interpersonal interactions, including LMX and TMX (Zhao and Cai 2021). The indirect effect of team members' EQ on team performance can be mediated by interpersonal interactions, including LMX and TMX (Zhao and Cai 2021). Moreover, two studies discussed the impact of a team leader's EQ on the transformational leadership (Hur et al. 2011), intra-team trust, and team performance (Chang et al. 2012). EQ training programs followed by participating in team-based learning improve team members' EQ (Clarke 2010a). Team flexibility (Günsel and Açikgöz 2013), and team culture (Jamshed and Majeed 2019) have been suggested as antecedents to the team's overall EQ.

In our covered studies, the research on the role of CQ in teamworking has only started since 2013, which is due to the fact that CQ is a recent concept in intelligence research. The results of the content analysis show that similar to EQ, CQ has been mostly treated as an independent variable, influencing various team processes and outcomes. However, in contrast to the studies included in the EQ cluster, the ones in the CQ cluster have been mostly performed at the individual level (Bogilović et al. 2017; Chen and Lin 2013; Henderson et al. 2018; Presbitero and Toledano 2018; Presbitero and Teng-Calleja 2019; Presbitero 2020a, b, 2021). Only two studies have been performed at the team level (Richter et al. 2021; Moon 2013), and three studies have a multilevel structure (Ali et al. 2019; Erez et al. 2013; Hu et al. 2019). Analysis of the relationships between team members' CQ and dependent variables have been focused on individual performance (Hu et al. 2019; Presbitero and Toledano 2018), knowledge sharing (Chen and Lin 2013), perceived team efficacy (Chen and Lin 2013), interpersonal process effectiveness (Presbitero 2021), communication accommodation (Presbitero 2021), and trust (Shaik et al. 2021). The results of these studies show a positive relationship between CQ and these dependent variables. The results also indicate that team-level CQ positively improves social integration and performance (Richter et al. 2021). Moreover, the results show that CQ can serve as a moderator, changing the nature of the relationship between a predictor (e.g., cultural diversity, cultural dissimilarity, foreign language anxiety) and various team processes and outcomes (Moon 2013; Presbitero 2020a, b; Henderson et al. 2018; Presbitero and Teng-Calleja 2019; Ali et al. 2019; Bogilović et al. 2017). Furthermore, training and learning opportunities (Presbitero and Toledano 2018; Erez et al. 2013), team-level trust (Erez et al. 2013), and communication norms (Henderson et al. 2018) have been demonstrated to improve the CQ of team members. The role of the leader's CQ has also been suggested as an important factor impacting the team's processes and outcomes (e.g., social integration and performance) (Presbitero 2020a; Presbitero and Teng-Calleja 2019; Richter et al. 2021).

Team-level analysis (Devine and Philips 2001; LePine 2003) and multilevel analysis (LePine 2005; Neuman and Wright 1999; Summers et al. 2012) have been used to structure the research on cognitive ability. The results of the content analysis show that team-level cognitive ability has been demonstrated to predict team performance (Neuman and Wright 1999; Devine and Philips 2001), and role structure adaptation (LePine 2003). Team members' cognitive ability, also, improve individual performance (Neuman and Wright 1999) and role structure adaptation (LePine 2005). Team performance has been also suggested to be indirectly influenced by both team-level cognitive ability (LePine 2003) and team members' cognitive ability (Summers et al. 2012).

To the authors' knowledge, this paper is the first that exceeds the scope of the existing review papers, which have so far been limited to a certain type of intelligence in a certain type of team. Focusing on publications between 1999 and 2021, there appears to be a shift of interest from cognitive ability to various other contemporary types of intelligence, such as EQ and CQ, which accounts for having more impact on team performance rather than cognitive ability does (Offermann et al. 2004). The results also show that there are only a few studies examining the different types of intelligence in a single study. In the articles included in our systematic literature review, three studies examined EQ and cognitive ability simultaneously. Only one study, discussed the effects of EQ and CQ, as well as EQ and social intelligence at the same time, indicating that only a small number of studies investigated multiple intelligences in the team context.

Moreover, the findings presented in this study show the growing interest in publications dedicated to GVTs in 2020 and 2021. The pace of research on GVTs has accelerated in recent years in response to globalization and the information technology revolution (Lepsinger and DeRosa 2010). Moreover, the COVID-19 pandemic has likely further increased the popularity of research in GVTs as organizations were forced to shift to remote working. Consequently, the importance of CQ, as individuals' ability to behave effectively in multicultural situations (Ang et al. 2007: 337), becomes evident.

### Future research directions

The results of the literature review show that there are some studies examining the role of different types of intelligence on team outcomes. Nevertheless, the number of such studies is relatively low. Thus, there is a need for further studies examining the role of various types of intelligence in different types of teams. As the results show, EQ has mainly been examined in face-to-face teams and often in a monocultural setting. However, recent research highlights the importance of studying EQ in GVTs (Davaei et al. 2022, in press). Extending the research to different types of teams and more international settings may provide fruitful avenues for future research.

Also, the results show that a limited number of studies investigated multiple intelligences in the team context. While intelligences are not necessarily single acts; instead, they are interactive and work together in a variety of ways (Martin 2001). Thus, a person can employ more than one intelligence in a given situation or behavioral act (Armstrong 1994; Green et al. 2005; Martin 2001). For example, recent research (e.g., Davaei et al. 2022, in press; Eberz et al. 2020) highlights the importance of EQ, in addition to CQ, in GVTs. Therefore, comparing the role of different types of intelligence in various types of teams could be a promising research area. Such studies may help to better understand the possible compensatory effects (Côté and Miners 2006) of the different types of intelligence and thus be of high relevance to both, theory, and practice.

Moreover, the results of the literature review show that the impact of team leader's different intelligences on various team outcomes has been discussed in a few studies. While prior research proposes that a team EQ and a leader EQ have a compensatory relationship in predicting team performance, meaning that either a high team EQ or a high leader EQ is sufficient to explain a high level of team performance (Chang et al. 2012). Future research may want to study the role of team leader's various types of intelligence in team processes and outcomes.

The findings also indicate that there has been more interest in determining the outcomes of, rather than the antecedents to intelligences. Determining the variables antecedent to intelligences, especially those improving intelligences in a teamwork setting can be an interesting topic for future research. This most likely holds for EQ and CQ. In contrast to an individual's cognitive ability which may be very difficult, if not impossible, to be improved (Coleman and Argue 2015), the findings suggest developing team members' EQ (Clarke 2010a) and CQ (Presbitero and Toledano 2018; Erez et al. 2013) by training programs and learning opportunities.

Although the majority of research in the domain of EQ (e.g., Jamshed and Majeed 2019; Lee and Wong 2017; Macht et al. 2019) and cognitive ability (e.g., Devine and Philips 2001; LePine 2003) are at the team-level, few studies have effectively lifted the level of analysis for the CQ above the individual level. That is due to the fact that CQ was originally conceptualized as an individual-level construct, and there is a lack of empirical research on the appropriate operationalization methods for CQ at the team level. Therefore, it is imperative for future research to consider team-level CQ. Moreover, as it has also been called by other researchers (e.g., Ashkanasy and Jordan 2008; Sharma and Hussain 2017), it is imperative for future research to try applying a multilevel perspective for EQ, CQ, and cognitive ability.

Table 6 presents a summary of the research gaps that were identified through our systematic literature review. It also outlines the key future research questions that can help address these research gaps.

**Table 6:** Future research agenda

	Research gaps	Future research questions
Emotional intelligence (EQ)	The need for extending the research on the impact of EQ on team processes and outcomes in face-to-face teams to other types of teams, such as multicultural teams, virtual teams, and GVTs	How does EQ impact team processes and outcomes in diverse team contexts, including multicultural, virtual, and global virtual teams?
	The need to investigate the impact of team leader's EQ on team processes and outcomes	What is the impact of team leader's EQ on team processes and outcomes in different types of teams and contexts?
	The lack of understanding regarding the variables that precede and enhance EQ in a teamwork setting	What are the antecedent variables that contribute to the development of EQ in a teamwork setting?
	The need for multilevel conceptualizations that considers the individual and team-level factors that contribute to the development and impact of EQ in teams	How do individual-level and team-level factors interact to influence the development and impact of EQ in teams, and how can this multilevel conceptualization be leveraged to enhance team processes and outcomes?
Cultural intelligence (CQ)	The insufficient exploration of the role of CQ in multicultural teams	How does CQ impact team processes and outcomes in multicultural teams?
	The lack of investigation into the potential influence of a team leader's CQ on team processes and outcomes	How does the team leader's CQ affect the team's processes and outcomes in multicultural teams?
	The need to explore the variables that precede and promote CQ in multicultural teams	What are the antecedent variables that contribute to the development of CQ in a multicultural team environment?
	The lack of studies that have investigated CQ at the team level	What is the relationship between CQ at the team level and team processes and outcomes, and how can team-level CQ be developed and leveraged to enhance team effectiveness in multicultural teams?
	The lack of a clear and comprehensive definition of the team-level CQ construct	How can we define and measure the construct of team-level CQ, and what are the implications of this construct for enhancing team outcomes in multicultural teams?
	The lack of multilevel conceptualizations that considers the individual and team-level factors that contribute to the development and impact of CQ in multicultural teams	How do individual-level and team-level factors interact to influence the development and impact of CQ in multicultural teams, and how can this multilevel conceptualization be leveraged to enhance team processes and outcomes?
Cognitive ability	The insufficient exploration of the role of cognitive ability in various types of teams	How does cognitive ability impact team processes and outcomes in various team contexts?
	The need to investigate the role of team leader's cognitive ability in various team processes and outcomes	What is the impact of team leader's cognitive ability on team processes and outcomes?
Multiple intelligences	The limited number of studies that have examined multiple types of intelligence, such as EQ, CQ, and cognitive ability, in a single study to explore their respective contributions to team processes and outcomes	How do different types of intelligence, such as EQ, CQ, and cognitive ability, interact and collectively influence team processes and outcomes? What is the relative importance of different types of intelligence in predicting team outcomes across diverse team contexts?

### Managerial relevance

This study extends our understanding of the role of different types of intelligence and their impacts on various team processes and effectiveness. Different types of teams (e.g., virtual, multicultural, etc.) provide both opportunities and challenges for organizations. However, various intelligences that individuals bring to a team, reduce the challenges of working in teams. For example, EQ has been suggested to facilitate effective communication in virtual teams (Pitts et al. 2012) and CQ can help multicultural teams reduce the obstacles arising out of cultural diversity (Scholz 2012).

The results of this study are primarily of interest to human resource managers dealing with team member selection and training. The findings suggest that organizations should integrate various intelligences into human resource processes depending on the team context. CQ and EQ include a set of malleable competencies and as such can be developed and trained through human resource interventions like cross-cultural and EQ trainings (Clarke 2010b; Coleman and Argue 2015; Earley and Ang 2003; Presbitero and Toledano 2018; Taras et al. 2013). Organizations can provide training and development programs to help employees improve their specific intelligences, thus, ultimately benefitting the collective goals of the team. In addition to that, using CQ and EQ assessment tools can help recruiters make better hiring decisions (Wasylyshyn 2010). This most likely also holds for IQ. Even though improving an employee's IQ may be very challenging, or even unfeasible (Coleman and Argue 2015), various IQ tests can be utilized to improve the selection process and aid hiring decisions. Therefore, when selecting new team members, various intelligence tests may be of help in finding the optimal team members.

### Limitations

This systematic review contributes to a better understanding of the domain of intelligences and its importance in the team. However, like all research, this study is not without limitations. The research approach chosen did not permit the inclusion of all research available on intelligences in teams. The research was limited only to two databases (WoS and EBSCO), and the search terms were only used in the title of articles. Moreover, the focus on the areas of business and management means that insight from only very specific research areas could be provided. While a large number of articles published on intelligences are in the area of psychology. Therefore, future research may want to expand the search by including more databases and various areas of research.

## Data Availability

The authors confirm that all data generated or analyzed during this study are included in this manuscript.
